# Research and performance analysis of random forest-based feature selection algorithm in sports effectiveness evaluation

**DOI:** 10.1038/s41598-024-76706-1

**Published:** 2024-11-01

**Authors:** Yujiao Li, Yingjie Mu

**Affiliations:** Harbin Normal University, Harbin, 150025 China

**Keywords:** Random forest, Feature selection algorithm, Sports big data, Sports data, Data statistics, Engineering, Mathematics and computing

## Abstract

The rapid progress in fields such as data mining and machine learning, as well as the explosive growth of sports big data, have posed new challenges to the research of sports big data. Most of the available sports data mining techniques concentrates on extracting and constructing effective features for basic sports data, which cannot be achieved simply by using data statistics. Especially in the targeted mining of sports data, traditional mining techniques still have shortcomings such as low classification accuracy and insufficient refinement. In order to solve the problem of low accuracy in traditional mining methods, the study combines the random forest algorithm with the artificial raindrop algorithm, and adopts a sports data mining method based on feature selection to achieve effective analysis of sports big data. This study is based on the evaluation method of motion effects using random forests, and uses feature extraction algorithms to study the motion effect impacts. It uses the information gain index to rank the importance of features and accurately gain the degree of influence of exercise on various indicators of the human body. Through simulation verification, the algorithm proposed by the research institute performs the best in accuracy and FI scores on the training and testing sets, with accuracies of 0.849 ± 0.021 and 0.819 ± 0.022, respectively, and F1 scores of 0.837 ± 0.020 and 0.864 ± 0.021, respectively. This indicates that the algorithm proposed by the research institute has high classification accuracy and performance proves that the Random Forest-based feature selection algorithm established in this study is superior to the existing traditional feature extraction and extraction methods in terms of both performance and accuracy. The proposal of this data analysis method has achieved accurate and efficient utilization of sports big data, which is of great significance for the development of the sports education industry.

## Introduction

With the rapid development of big data technology and artificial intelligence, the data analysis capabilities in the sports field have been unprecedentedly improved^1^. Sports performance evaluation, as an important branch of sports science, mainly studies the impact of sports on human health, physical fitness improvement, and sports performance^2^. In this process, how to evaluate the effectiveness of sports through scientific and quantitative methods has become one of the core issues in sports research. Through the evaluation of exercise effectiveness, researchers can comprehensively understand the training status, physical function response, and improvement of various indicators of the body after long-term training of athletes^3^. This provides reliable data support for optimizing training plans and improving athlete performance. However, traditional sports performance evaluation methods mostly rely on empirical judgment or basic statistical methods, and fail to fully explore the deep level information in sports data^4^. This has led to low classification accuracy and low data utilization efficiency of existing methods when processing complex motion data, which cannot provide effective support for decision makers and trainers. With the rapid growth of sports big data, how to extract key features from a large amount of complex sports data in order to accurately evaluate the impact of sports on the human body has become an urgent technical challenge^5^. Existing research has shown that traditional data mining methods face problems such as low classification accuracy and inability to effectively handle high-dimensional data when dealing with multi-dimensional and nonlinear motion data^6^. This not only limits the practical application value of the data, but also makes it difficult to provide effective feedback for coaches and athletes. Therefore, an innovative sports data mining method combining optimized Artificial Raindrop Algorithm (ARA) and Random Forest Algorithm has been proposed. In the random forest algorithm, by weighting the features, the algorithm can quickly identify key features, thereby improving classification efficiency. At the same time, utilizing the reverse learning mechanism, the ARA algorithm was optimized to automatically adjust motion parameters, thereby enhancing the model’s ability to recognize and classify motion features. The research aims to improve the accuracy and performance of existing sports data mining methods through innovation in these two aspects, providing new ideas and tools for big data development in the sports education industry.

There are two innovative points in the research: the first point is the introduction of the idea of weight allocation, which weights the features when selecting them in the random forest algorithm, thereby helping the algorithm lock in key features more quickly; The second point is to study the introduction of reverse learning mechanism to optimize the artificial raindrop algorithm. The optimized artificial raindrop algorithm through reverse learning mechanism can achieve automatic adjustment of motion parameters, thereby improving the model’s ability to recognize and classify motion features. This research has four parts. Part 1 provides an overview of the research background and summarizes the research in related fields. Part 2 describes the feature extraction algorithm introducing RF. Part 3 validates the feature extraction algorithm introducing RF and experimentally verifies the results of its motion effect evaluation. Part 4 summarizes and outlooks the whole research.

## Related works

Sports effect assessment is an essential research direction in sports science. The growth of data science and ML technology has led more and more scholars to apply ML-based algorithms to motion performance evaluation. Among them, the RF algorithm, as a powerful ML tool, is also widely used in this field.

Narasimhulu in the proposed work used dragonfly algorithm to implement the design and training of random decision forest classifier and selected features. Renal disturbances in ultrasound scans are identified and classified by providing some substantial content descriptive parameters. A series of quantitative features are synthesised for each image and principal component analysis is performed to minimise the features to generate a set of wavelet-based multi-scale features^7^. Herrera-Semenets et al. proposed a novel feature selection algorithm for intrusion detection scenarios. The new multimetric algorithm was designed which reduced the training dataset dimensionality, and was extensively tested by various datasets. The algorithm has higher efficacy compared to other algorithms for intrusion detection^8^. Four different ML algorithms were used by Lovric et al.) and combined with multilevel replacement importance feature selection and Bayesian hyperparameter optimisation to predict solubility based on the chemical structure information, resulting in a ranked score for selecting the best model. The ranking score is a weighted combination of generalisation, number of features and test performance with the best predictive and generalisation capabilities^9^. Adamichou et al. analysed deconvolutional classification criteria and non-criteria features for clinical selection of systemic lupus erythematosus. Feature selection and model construction were performed using RF and Least Absolute Shrinkage and Selection Operator-Logistic Regression (LASSO-LR) techniques. The tool generated probabilities of SLE risk that were positively correlated with the severity of the disease and organ damage. It also enabled unbiased classification of the validation cohort based on the likelihood of SLE compared to other diagnoses^10^. Pan et al. compiled a dataset of 31 alloy sequences and 179 high GFA alloys. They collected the alloys from peer-reviewed publications and used regression to RFs to predict a missing but necessary feature in the dataset. The optimization of hyper-parameters, the quantity and independence of input features and the appropriate model did well in obtaining good GFA prediction results^11^.

In studies related to the assessment of the effects of sport, the performance of athletes in training and competition is assessed by objective and quantitative means. Amatriain-Fernández et al. calculated the comprehensive information and magnitude of inhibitory control benefits for healthy children and adolescents (HC-As). They conducted a random effects analysis on 3 variables: accuracy, response time, and comprehensive score. HC-As showed only slight improvement in inhibitory control after participating in various chronic exercise interventions. The strict inclusion criteria and highly variable nature of exercise intervention design may have a negative impact on the results^12^. Liu et al. constructed a multimedia assisted teaching effectiveness evaluation model based on random forest algorithm, optimized teaching quality evaluation indicators, analyzed the level of online teaching in physical education in universities, and completed the evaluation of multimedia assisted teaching effectiveness in physical education courses. The research results have demonstrated the effectiveness of the model, with user satisfaction reaching 72%, which helps to improve teaching quality and efficiency, and promote scientific teaching management^13^. Chen designed an intelligent fuzzy system model to improve the accuracy of teaching effectiveness evaluation. The evaluation index system was adjusted through discussions with experts, and the Analytic Hierarchy Process was used to determine the weights of the indicators. The single-layer and overall rankings of the indicator matrix were then calculated for evaluating physical education courses. The research results show that the fuzzy evaluation accuracy of the model exceeds 95.63%, demonstrating high evaluation performance and strong practicality^14^. Yuan has developed a sports training decision support evaluation system that combines data mining techniques to meet the scientific training needs of athletes. The system analyzed the software running characteristics and focused on the processing of association rule data in sports evaluation, introducing an improved Apriori algorithm to enhance the evaluation effect. Experimental results have shown that this method can effectively assist in decision-making for sports training^15^. Zang proposed an extended training evaluation model based on neural networks, aimed at providing reference parameters for both trainers and trainees. The model first analyzes different extension projects and classifies them, then extracts key indicators from the learning process as features, and finally evaluates them using neural networks. The research results have confirmed the effectiveness of this method^16^.

In summary, various types of computational simulation methods based on RFs have been applied in many different fields for feature extraction. And many scholars have taken different methods to practice and verify in sports effect evaluation. The purpose is to discuss the current research status, methodology, application and performance analysis of RF-based feature selection algorithms (RF-FSA) in sports effect evaluation, with a view to providing references and insights for future research.

## RF-FSA and optimised ARA for sports effectiveness evaluation

A RF-based exercise effect evaluation method is studied, which does not depend on the selection of models or the assumption of conditions, thus improving the classification accuracy of exercise behaviour. On this basis, a new inverse learning method is proposed, and on this basis, an artificial raindrop algorithm (ARA), which combines inverse learning, is introduced to modify the parameters of the “weight reduction” exercise.

### RF-FSA and its application to sport effect assessment

**Fig. 1:**
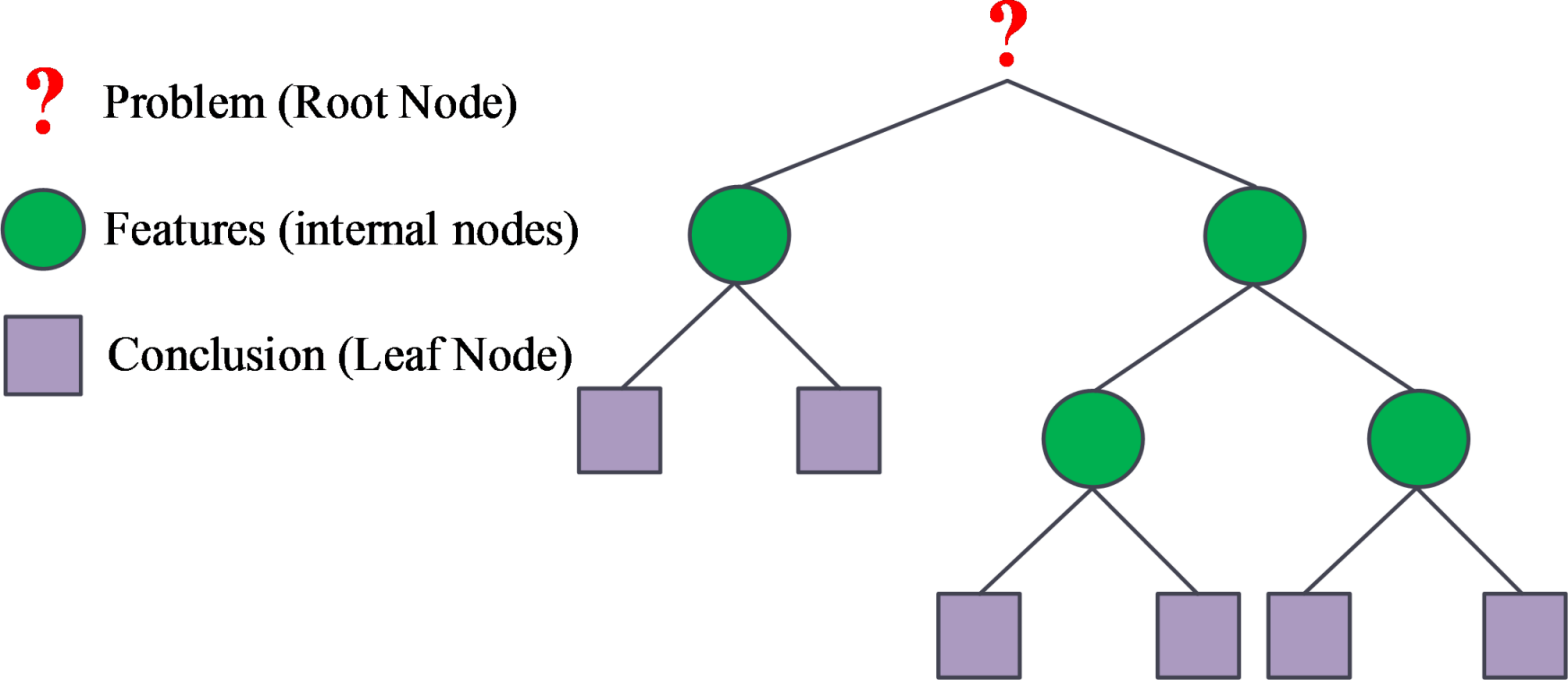
Decision tree diagram.

RF is a DT-based method. When constructing a DT, self-organised resampling is used to select the features of the sample, and finally multiple DTs vote on it^17,18^. RF is able to handle high-dimensional data. Additionally, it also has some noise suppression function, its performance is relatively stable and easy to implement. DT usually includes three parts: problem, features and conclusion. In the DT decomposition, usually use the random selection method to select a number of features to be selected, and its purity calculation, the use of feature purity measure its strengths and weaknesses, and the use of purity to determine its divisibility characteristics. The DT schematic is Fig. 1.

**Fig. 2:**
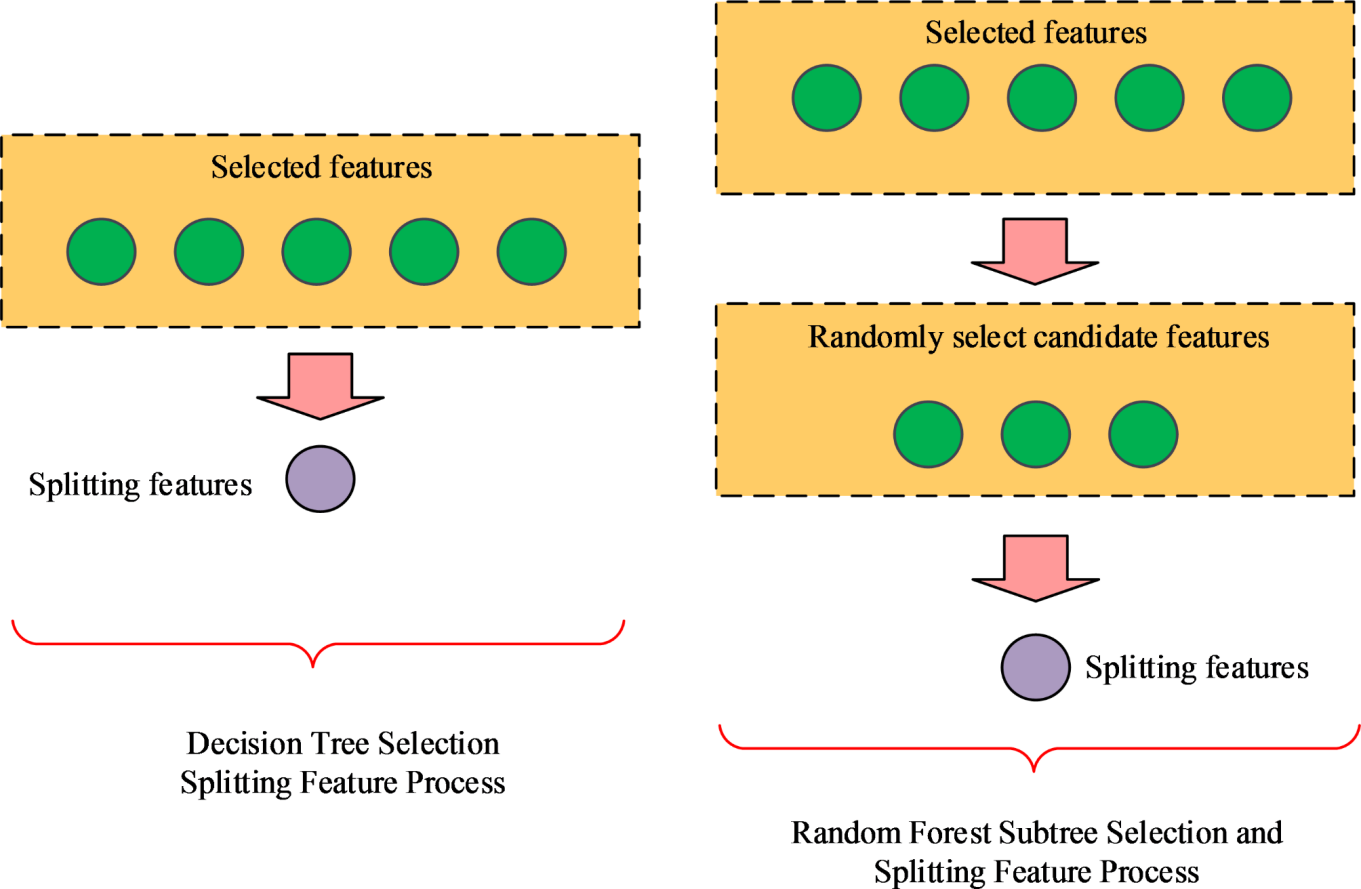
Comparison of DT and RF sub-tree selection splitting features.

In Fig. 1, it can be seen that unlike the traditional method of constructing a single decision tree, the decision tree in a random forest first randomly extracts a portion of the features to be selected when extracting separable features. In order to highlight the differences in the features extracted from multiple decision trees in the random forest algorithm, the idea of weight allocation was introduced to assign weights to the outputs of different decision trees. The allocation of weights adopts a weight matrix, which can be convolved with the output results to obtain weighted important features. Then the algorithm selects the best feature from the weighted features and uses it as a separable feature. Then randomly extract separable features from the features to be selected, and then randomly select separable features from these subtrees^19,20^. For each node, the feature with the highest information gain is selected for node partitioning by calculating the information gain of all candidate features. In this way, RF can select the most useful features for classification from high-dimensional data. The comparison of DT and RF sub-tree for selecting split features is Fig. 2.

In Fig. 2, a set of data samples is built by using the indicators related to the action effect in the database, which are the data features, and the corresponding attributes are filtered, based on which they are labelled, and partitioned into a training and a test set, and each feature is regarded as a node, and the information entropy is utilized to measure the purity of the samples, and the information entropy is expressed by the formula as shown in Eq. (1).

1 $$Ent(X)= - \sum\limits_{{i=1}}^{n} {{p_i}\log {p_i}}$$

In Eq. (1), ${p_i}$ is the proportion of the current sample set $X$ in the $q$ class of samples, and the smaller the value of$Ent(X)$, the higher the purity of the sample $X$. Subsequently, the criterion of information gain (IG) is used to select the optimal features, defined as shown in Eq. (2).

2 $$g(X,A)=Ent(x) - Ent(X\left| A \right.)$$

In Eq. (2), $g(X,A)$ is the IG of feature $A$ in sample $X$ . In the random forest algorithm, feature selection is achieved through information gain. RF is a classification method consisting of multiple trees. For this reason, the study proposes a DT algorithm based on RF. For a particular feature, the amount of information it can provide changes when the feature is used and when it is not used, and the discrepancy between the B&A amount of information is the provided features. Its information is called entropy and it is used to measure the uncertainty of a random variable. Assuming a finite discrete random variable with $n$ values and the probability of the first $i$ value is ${p_i}$, the random variable entropy is defined as Eq. (3)

3 $$H= - \sum\limits_{{i=1}}^{n} {p({x_i}){{\log }_2}p({x_i})}$$

In classification and clustering problems, when the category in which the document is in changes more, it contains more information, so the IG given by feature A to classification C or clustering C is Eq. (4).

4 $$IG(A)=H(A) - H(C\left| A \right.)$$

DTs, as one of the most basic classification methods, usually classify features into two types. The DT algorithm classifies each feature until it is classified into two categories. In this method, we use an information increment to check whether a feature generates a node or not. In each DT, the related out-of-the-box value is chose to calculate its prediction error rate. Based on this, random noise is added and the forecast error is analysed to filter out meaningful features. The IG method is used to remove a certain percentage of features at a time to get a new set of attributes, as expressed in Eq. (5).

5 $${a_*}={\arg _{a \in A}}\hbox{max} Gain(D,a)$$

The study’s precision is calculated using the experimental evaluation criterion of top@k precision. This measures the proportion of human data obtained by the algorithm that affects mood. Higher precision means the more efficient. The formula for precision is Eq. (6).

6 $$\Pr ecison=\frac{k}{n}$$

In Eq. (6), $k$ denotes the impact indicators matching the real situation, and $n$ denotes the selected overall indicators in the real situation. However, the information increase only considers the role of an attribute on the overall classification system, and cannot refine a class, so it can only discriminate the same set of features, and cannot achieve the set of features with differences between different classes. Regularisation is a common model optimisation method, which can reduce the complexity and solve the problem of over-fitting, the expression of which is shown in Eq. (7).

7 $$\bar {J}(w;X,y)=J(w,X,y)+\alpha \Omega (w)$$

In Eq. (6), $X$ and $y$ are the training samples and labels, $w$ is the vector of weight coefficients, $J()$ is the empirical risk, $\Omega (w)$ is the regularisation term, $\alpha$ is the strength of the controller’s regularisation, and the commonly used $\Omega$ functions are the paradigms ${l_1}$ and ${l_2}$, and the corresponding regularisations are ${l_1}$ regularisation and ${l_2}$ regularisation. ${l_1}$ The mathematical expression of regularisation is shown in Eq. (8).

8 $$\bar {J}(w;X,y)=J(w,X,y)+\lambda {\left\| w \right\|_1}$$

${l_2}$The mathematical expression for regularisation is shown in Eq. (9).

9 $$\bar {J}(w;X,y)=J(w,X,y)+\alpha {\left\| w \right\|_2}$$

Combining the advantages and disadvantages of ${l_1}$ and ${l_2}$ regularisation, the elastic net pyrrole emerges with the digitisation concept shown in Eq. (10).

10 $$\bar {J}(w;X,y)=J(w,X,y)+\alpha \lambda {\left\| w \right\|_1}+\frac{{1 - \alpha }}{2}\alpha {\left\| w \right\|_2}$$

The importance of features is evaluated by using RF method, mainly by evaluating the importance of each feature and then using Gini coefficient (Gini) to calculate the importance of these features, where a smaller value of Gini indicates that it is more pure and the importance of these features is greater^21–23^. The Eq. (11) calculates the Gini index.

11 $$Gin{i_m}=\sum\limits_{{k=1}}^{{\left| {\left. k \right|} \right.}} {\sum\limits_{{k \ne k}} {{p_{mk}}{p_{mk^{\prime}}}} } =1 - \sum\limits_{{k=1}}^{k} {{p^2}_{{mk}}}$$

In Eq. (11), $p_{{mk}}^{2}$ represents the proportion of category $k$ in node $m$ , the importance of feature ${X_j}$ in node $m$ . The change amount in Gini index B&A branching of node $m$ is Eq. (12).

12 $$VIM_{{jm}}^{{Gini}}=Gin{i_m} - Gin{i_l} - Gin{i_r}$$

In Eq. (12), $Gin{i_l},Gin{i_r}$ denotes the Gini indices of the 2 nodes after branching, and the importance of ${X_j}$ in the first $i$ tree is Eq. (13).

13 $$VIM_{j}^{{Gini}}=\sum\limits_{{m \in M}} {VIM_{{ij}}^{{Gini}}}$$

Assuming that there are $n$ trees in the RF, the importance of ${X_j}$ at the $i$ th tree is Eq. (14).

14 $$VIM_{j}^{{Gini}}=\sum\limits_{{i=1}}^{n} {VIM_{{ij}}^{{Gini}}}$$

Finally, all the feature importance scores were normalised. For the more important features that affect the movement effect, the Pearson correlation coefficient (PCC) is used for the similarity calculation of the features, and the PCC ${\rho _{X,Y}}$ is expressed as shown in Eq. (15).

15 $${\rho _{X,Y}}=\frac{{\operatorname{cov} (X,Y)}}{{{\sigma _X}{\sigma _Y}}}$$

**Fig. 3:**
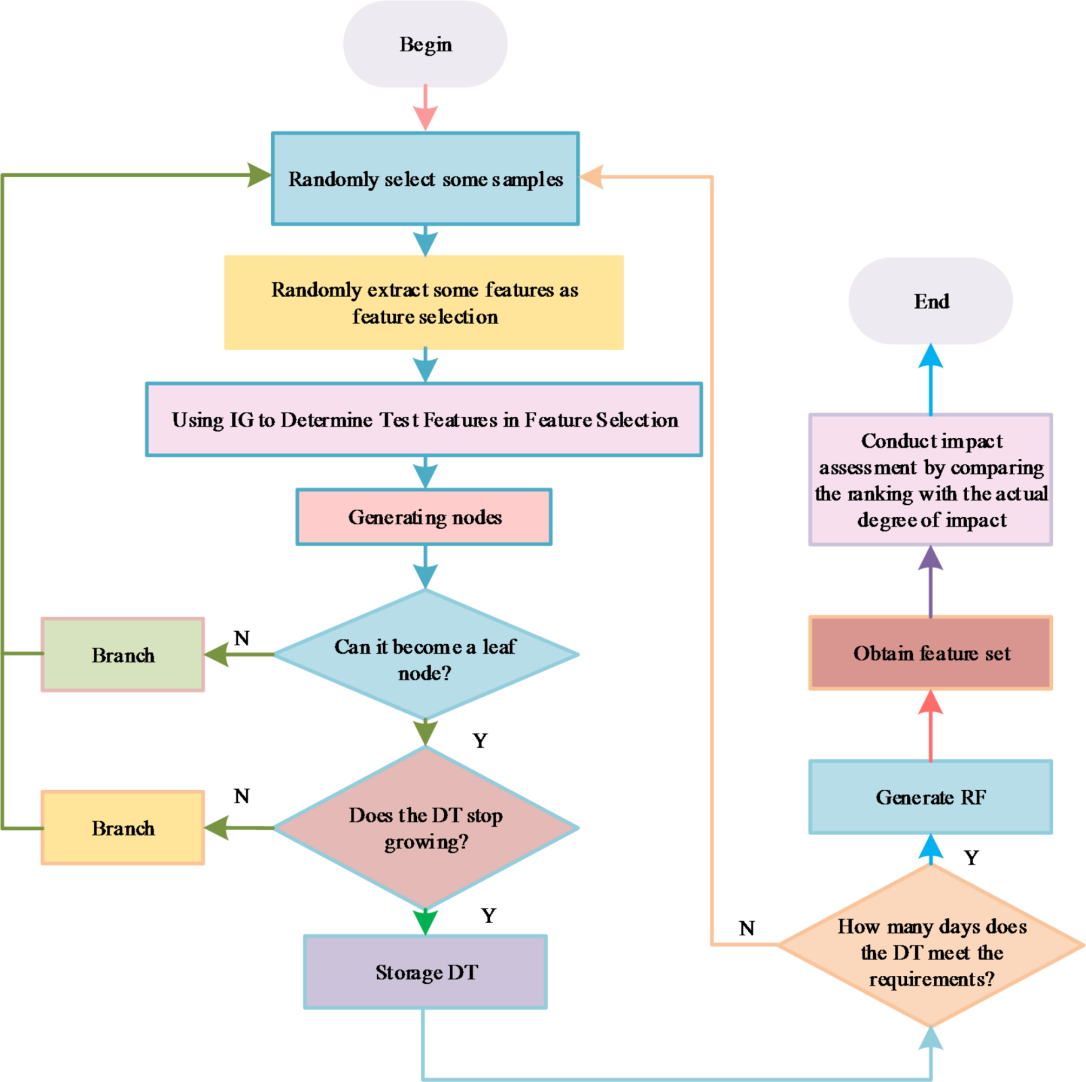
Schematic diagram of RF evaluation algorithm.

In Eq. (15), $\operatorname{cov} (X,Y)$ is the variance of $X,Y$. $\sigma$ denotes the standard deviation. The study takes the two types of competition and non-competition as samples, constructs the sports effect evaluation algorithm, obtains the intensity of the action of competition type sports on each index of the organism, and then evaluates its sports effect, as shown in Fig. 3.

In Fig. 3, the above algorithm is also used in the study of other types of sports activities, and finally the set of attributes with significant influence on the physical fitness index is obtained for each type of folk sports activity. By analysing and reasoning about the obtained set of attributes of indices with significant influence, the status of the influence of the corresponding sport on the body mass index is derived. The calculated influence of exercise on the BMI was compared with the Ground-Truth method and the algorithm was validated on this basis.

### Evaluation and analysis of the ARA optimised based on the reverse learning mechanism for the evaluation of movement effects

The study proposes ARA based on opposition-based learning (OBL + ARA) to achieve automatic adjustment of motion parameters. The reverse learning mechanism is an effective strategy in heuristic optimization algorithms to accelerate convergence and enhance the diversity of solutions. In the artificial raindrop algorithm, the reverse learning mechanism generates the reverse points of the current solution and explores more extensively in the solution space, helping the algorithm approach the global optimal solution faster. For a certain component of an unknown solution in the search space, the definition of its inverse point is shown in Eq. (16).

16 $${c^{'}_{i}}=b_{i}+d_{i}-c_{i}$$

In Eq. (16), ${c^{'}_{i}}$ is the $i$ -th component of the current solution, and $d_{i}$ and $b_{i}$ are the minimum and maximum values of the $i$ -th dimension in the search space, respectively. During each iteration, the artificial raindrop algorithm evaluates the fitness values of both the current solution $c$ and its inverse point ${c^{'}}$ simultaneously. Set the objective function as $f(c)$ . In the evaluation, if the fitness of the reverse point is better than the current solution, the reverse point is selected as the new solution. By calculating the fitness of the current solution and the reverse point, select the solution with better fitness, as shown in Eq. (17).

17 $$c=\left\{ \begin{array}{ll}\!{c^{'}},f({c^{'}})<f (c) \\\! {c,f({c^{'}})\geq\;f(c)}\end{array}\right.$$

Through Eq. (17), the reverse learning mechanism can enable the algorithm to explore more extensively in the solution space, thereby increasing the likelihood of finding the global optimal solution. In order to further enhance the flexibility of the algorithm, the artificial raindrop algorithm introduces an adaptive adjustment mechanism. This mechanism dynamically adjusts the application frequency and intensity of reverse learning based on the iterative process, mainly achieved through adaptive flow coefficients. The update formula for the adaptive flow coefficient is shown in Eq. (18).

18 $$\beta_{t+1}=\beta_{t}\times(1-\frac{t}{T})$$

**Fig. 4:**
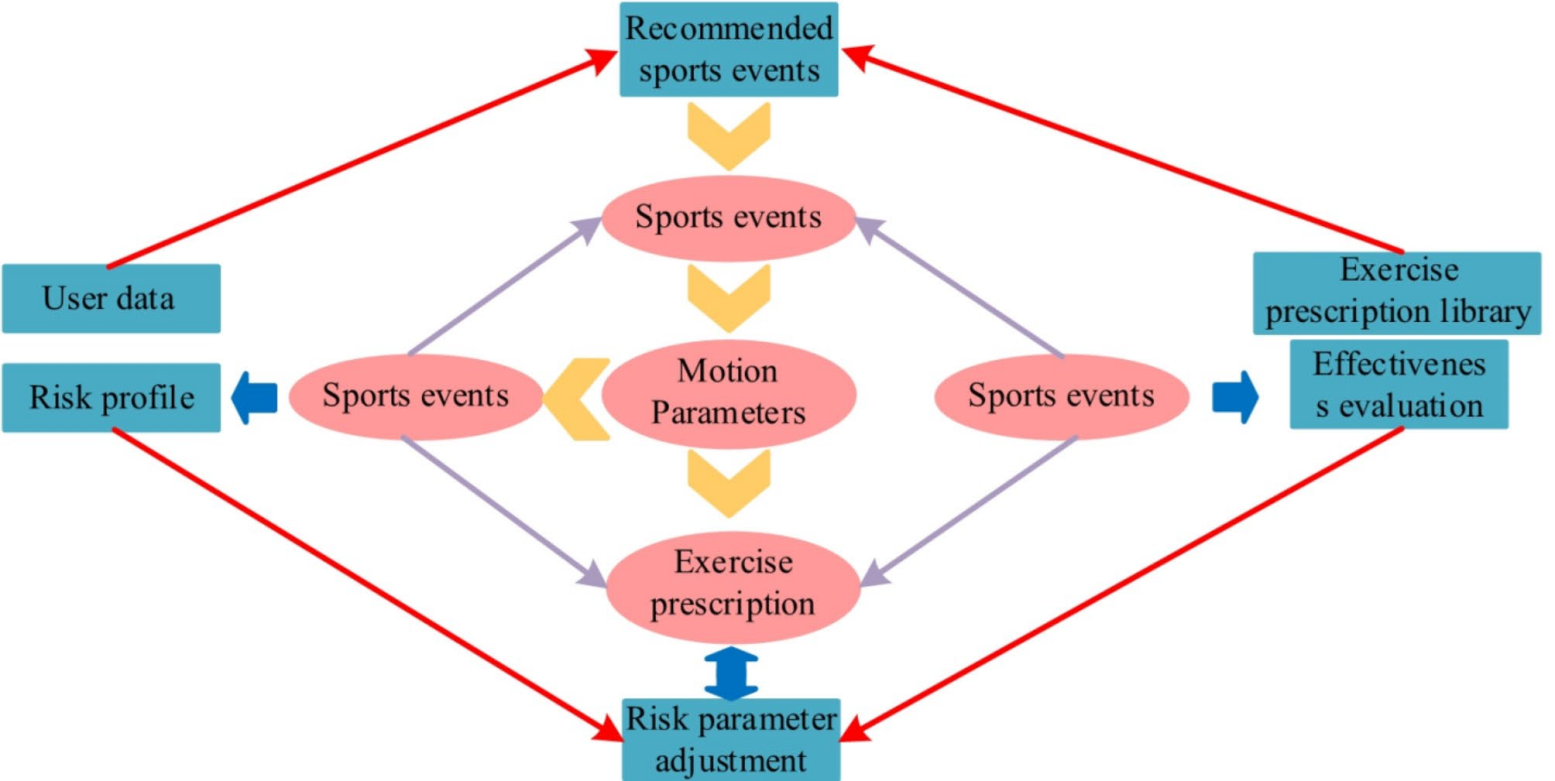
Technical roadmap diagram.

In Eq. (18), $\beta_{t}$ represents the adaptive flow coefficient in the $t$-th iteration, and $T$ represents the maximum number of iterations. As the number of iterations increases, the flow coefficient gradually decreases, allowing the algorithm to transition from global search in the initial stage to local search in the later stage, achieving optimization balance. The artificial raindrop algorithm can perform more effective searches in the solution space, utilizing reverse learning mechanisms and adaptive adjustments to improve the global search capability and convergence speed. The technical route of the model is shown in Fig. 4.

**Fig. 5:**
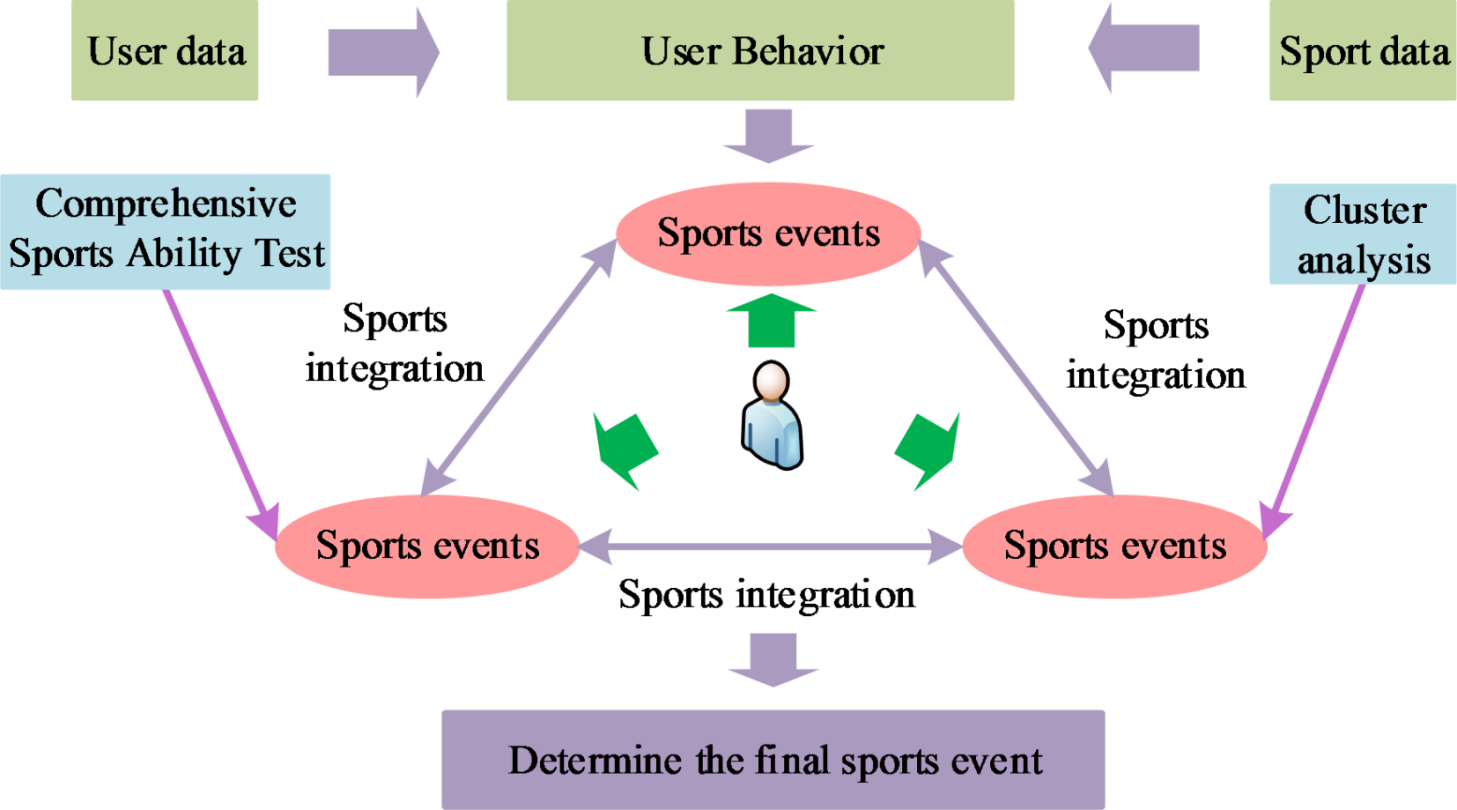
Process of determining sports events.

In Fig. 4, in the case of long rainfall retention time, retrograde learning is used to guide the retention rainfall out of the retention area in reverse; on this basis, the existing search interval is adaptively adjusted by using the difference of raindrop potential energy to improve the diversity of the population and the convergence rate. In the optimisation process, the dispersion of the global solution set in the target space is appropriately increased by introducing adaptive flow coefficients to achieve a better equilibrium effect. The study establishes the comprehensive athletic ability level of the athletes based on the evaluation indexes and corresponds to the standard exercise prescription to form the set of action items sorted according to the athletic ability. Finally, three different action sets are combined to get the final action items. The specific process of deciding the sport items is Fig. 5.

**Fig. 6:**
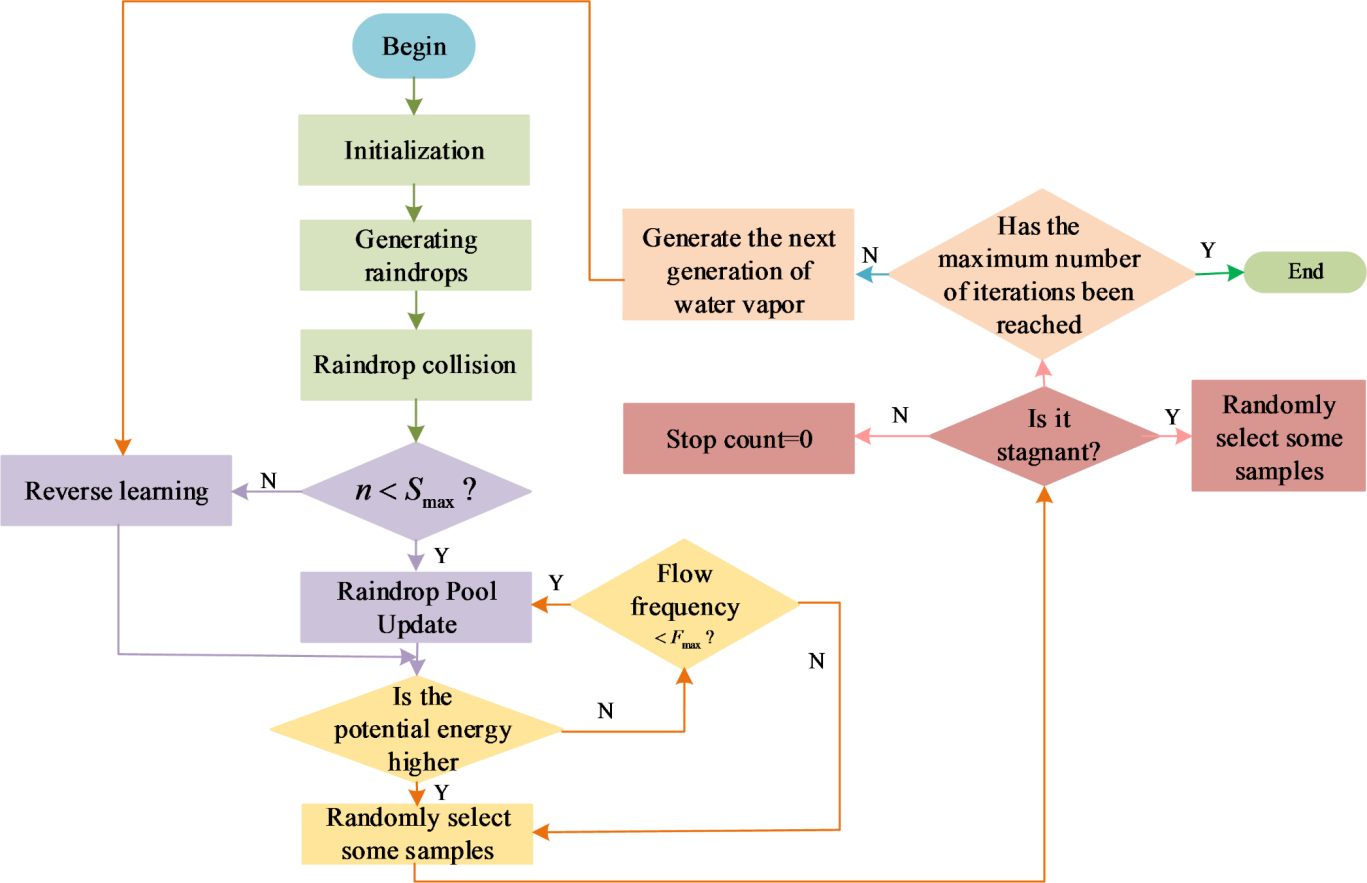
The flow chart of OBL + ARA.

In Fig. 5, it can be seen that the process of determining the sport items is divided into three parts, which are determined from three aspects, namely, user data, sport data and user behaviour analysis; user data is mainly used to determine the level of the sport ability through the comprehensive sport ability test, and the sport data is mainly used to determine the effect similarity through the clustering analysis, and the final combination of the user’s preference, the sport ability and the running effect is combined to determine the final of the exercise programme. In the ARA because the size of the raindrop pool is fixed, the more iterations of the raindrop pool, the fuller the raindrop pool will be. The algorithm employs a new computational method, i.e., by converting the rainwater in the raindrop pool into a raindrop pool with minimum potential energy, so that the water droplets in the raindrop pool introduce the rainwater mass in the raindrop pool into the water mass with less potential energy. The flow of the implementation of the OBL + ARA algorithm is shown in Fig. 6.

In Fig. 6, the study proposes a bubble sorting-based rainwater bank update algorithm, which sorts the rainwater banks in the rainwater bank according to the size of the potential energy, removes the largest rainwater bank among them, and adds the rainwater bank with the lowest current potential energy to realise the updating of the rainwater bank. On this basis, a random sampling method based on Gaussian perturbation is proposed, which can effectively improve the algorithm’s optimality seeking ability and reduce the problem that falling into the local-optimum easily, and the maximum value of stagnation time is set to 10, the maximum value of flow rate is set to 3, and the stagnation time, the rainwater pool and the water vapour are initialised.

## Analysis of RF-FSA and ARA in sport effectiveness evaluation

In order to evaluate the role of RF feature extraction algorithm and ARA in the evaluation of the effectiveness of sports, the study explores the trend and correctness of the model by adjusting the parameters such as the maximum number of features, the maximum tree depth, and the number of subtrees.

### Comparison of parameter trends in RF feature extraction algorithms and accuracy across multiple sports

The study used a comparative method to design a performance analysis experiment for the model. The experimental device used was a computer equipped with a Core i7-12700KF processor, 16GB of running memory, and a GTX1080 graphics card. The system environment is Windows 10, the data analysis model is constructed using JAVA voice, and the compiler is Eclipse. The dataset used by the research institute comes from the sports department of a certain university, covering data from multiple sports such as track and field, basketball, swimming, etc. The dataset for each project includes basic physical fitness indicators of participants, such as height, weight, heart rate, and multiple exercise performance data. The dataset has a total of 1500 participants, with 70% used for training and 30% for testing. In data preprocessing, the missing data was first processed by filling in the average value. For outliers, the principle of 3 times the standard deviation was adopted for removal. In the experiment, key parameters of the random forest, such as the number of decision trees and maximum tree depth, were optimized through grid search. Specifically, the number of decision trees is adjusted within the range of [50, 100, 150, 200], and the maximum tree depth is adjusted within the range of [10, 20, 30, 40]. Each parameter combination is evaluated for model performance using 5-fold cross validation to ensure the robustness and generalization ability of the model.

**Fig. 7:**
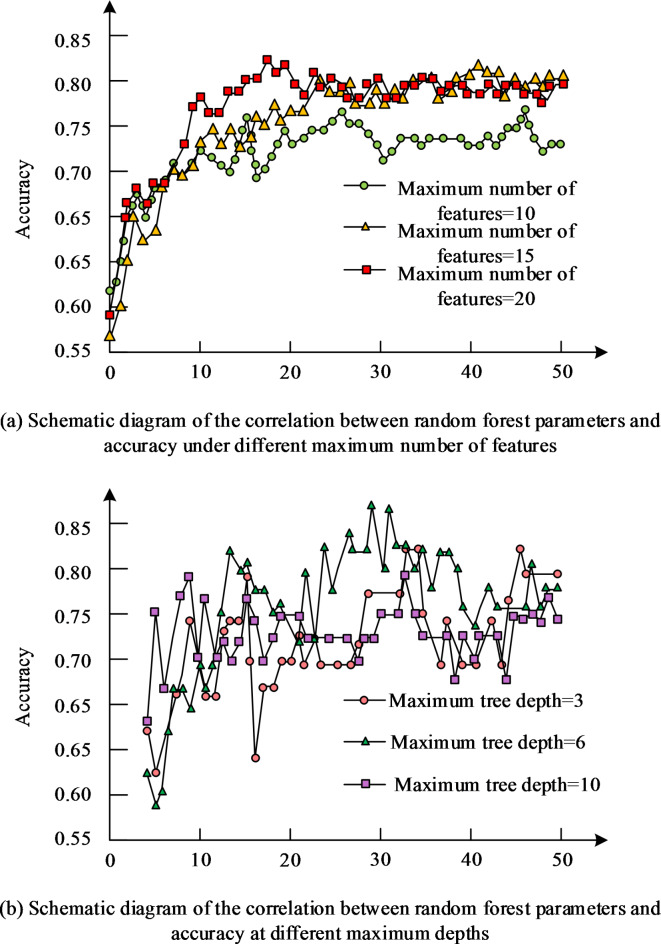
Schematic diagram of the correlation between RF parameters and accuracy.

The study used the trained model for accuracy testing. The experiment evaluated the impact of each parameter on the model through accuracy evaluation. The trend obtained is shown in Fig. 7.

**Fig. 8:**
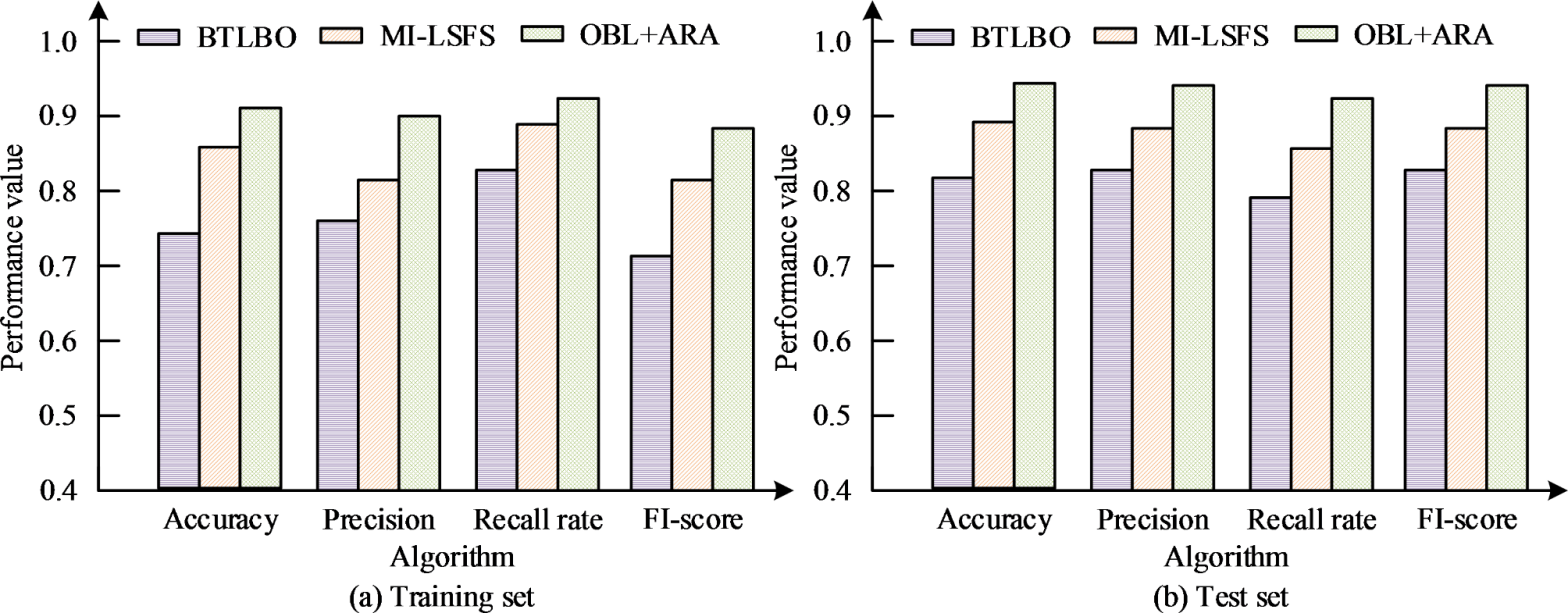
Comparison chart of experimental mean results.

In Fig. 7, the correct rate of the model increases with the increase of the sub-trees, and the recognition rate can be improved by increasing the features and the max-tree depth as well, but the number of 15 and 20 features does not have a great influence on the recognition rate, and the maximum tree depth is too large to lead to a decrease in accuracy. Select the Binary Teaching Learning Based Optimization (BTLBO) algorithm, Multiple Instance Local Salient Feature Selection (MI-LSFS) algorithm, and the OBL + ARA algorithm proposed by the research institute for performance comparison testing. The accuracy, recall, precision and F1-score of prediction were utilized to evaluate the strengths and weaknesses, and the experimental means are displayed in Fig. 8.

Figure 8 shows the comparison of three algorithms on four performance indicators. Figure 8 (a) shows the test results on the training set, where the OBL + ARA model performs the best in accuracy, precision, and F1 score, all exceeding 0.9. The MI-LSFS model performs the best in recall rate, close to 1, but has the lowest on FI score, about 0.75. In contrast, the BTLBO model has slightly lower accuracy than OBL + ARA, and performs equally poorly in terms of precision and recall. In Fig. 8 (b), the test results of the test set show that the BTLBO model has the highest accuracy, close to 0.85, while its accuracy is slightly lower than OBL + ARA, close to 0.85. The OBL + ARA model has the highest accuracy on the test set, close to 0.9, but slightly lower than BTLBO in terms of recall. The MI-LSFS model has the lowest accuracy on the test set, below 0.8. On FI score, the OBL + ARA model still maintains the highest value, around 0.85. The FI score of BTLBO and MI-LSFS models are similar, above 0.85 and 0.8, respectively. The results indicate that OBL + ARA performs well in processing training data, while BTLBO has a slight advantage on the test set. Although MI-LSFS has a high recall rate on the training set, it performs poorly overall on the test set. Next, a comprehensive performance comparison of the algorithms was conducted, and the results are shown in Table 1.

**Table 1:** Comparison of experimental mean variance between BTLBO, MI-LSFS algorithm, and OBL + ARA algorithm.

Algorithm	Dataset	BTLBO	MI-LSFS	OBL + ARA
Accuracy	Training set	0.846 ± 0.024	0.716 ± 0.023	0.849 ± 0.021
	Test set	0.815 ± 0.025	0.796 ± 0.024	0.819 ± 0.022
Precision	Training set	0.806 ± 0.025	0.824 ± 0.025	0.833 ± 0.019
	Test set	0.815 ± 0.023	0.834 ± 0.023	0.854 ± 0.020
Recall rate	Training set	0.845 ± 0.023	0.849 ± 0.023	0.855 ± 0.021
	Test set	0.783 ± 0.024	0.746 ± 0.022	0.788 ± 0.019
FI-score	Training set	0.811 ± 0.023	0.755 ± 0.023	0.837 ± 0.020
	Test set	0.854 ± 0.024	0.812 ± 0.022	0.864 ± 0.021

In Table 1, it can be seen that the overall accuracy of the OBL + ARA algorithm is the highest, with results of 0.833 ± 0.019 and 0.854 ± 0.020 in the training and testing sets, respectively. Next is MI-LSFS, with BTLBO having the lowest accuracy. This indicates that the OBL + ARA algorithm has higher accuracy in identifying positive samples. In the training set, OBL + ARA and BTLBO algorithms exhibit similar high accuracy, while MI-LSFS has significantly lower accuracy. In terms of recall rate, OBL + ARA still has the highest recall rate, at 0.855 ± 0.021. The other two algorithms are lower than this algorithm and have no significant difference. In addition, the FI score of OBL + ARA is 0.837 ± 0.020, followed closely by BTLBO, while MI-LSFS has the lowest FI score. In the test set results, the accuracy of OBL + ARA was also significantly higher than the other two comparison algorithms, at 0.819 ± 0.022. However, compared to the training set, there is a slight decrease, indicating that the model may have some overfitting on the test set. In terms of recall index, the recall rate of OBL + ARA is 0.788 ± 0.019, and the recall rate of BTLBO is 0.783 ± 0.024, with a small difference between the two, while MI-LSFS has the lowest recall rate. OBL + ARA has a slight advantage in identifying all positive samples. In terms of FI score, the difference between OBL + ARA and BTLBO results is also relatively small, at 0.864 ± 0.021 and 0.854 ± 0.024, respectively, while MI-LSFS remains the lowest. Overall, the OBL + ARA algorithm performs well in most metrics, especially in accuracy and F1 score, indicating that the OBL + ARA algorithm can learn data features well during the training phase. Moreover, the OBL + ARA algorithm performs exceptionally well in all four metrics, particularly in terms of accuracy and F1 score. Although the BTLBO algorithm has a small gap in recall rate compared to OBL + ARA, it is slightly inferior in other indicators. However, the MI-LSFS algorithm performs relatively poorly in all metrics, especially in accuracy and F1 score, where its performance is significantly inferior to the other two algorithms. The results show that the OBL + ARA algorithm has higher stability and accuracy in processing feature selection and motion effect evaluation, and is the algorithm with the best comprehensive performance among these three algorithms. The experiment was compared with the use of mean and variance, where the training and testing sets are set up in the similar way. Table 2 shows the specific data.

Table 2 shows the performance of three algorithms, BTLBO, MI-LSFS, and OBL + ARA, in four different task categories: wrestling, competition, skill, and modern school. By comparing their performance in top@3 the top@5 and top@10 evaluate performance based on the mean and variance of the situation. In wrestling tasks, OBL + ARA performs the best in all evaluation metrics, especially in top@3 and top@10 in this case, the results were 0.66 and 0.8, respectively, significantly higher than BTLBO and MI-LSFS. In competitive tasks, OBL + ARA also performed outstandingly top@3 and top@5 in this case, the results were 0.7 and 0.7 respectively, with BTLBO performing the worst in this category. In skill based tasks, OBL + ARA top@3 the top@5 and top@10 the results under the given conditions are 0.6, 0.7, and 0.7, respectively, which are significantly better than other algorithms. Finally, in modern school tasks, the performance values of OBL + ARA are top@3 the top@5 and top@10 in the cases of 0.66, 0.7, and 0.8 respectively, they also show significant advantages. Overall, in all categories and top@N under the setting, the OBL + ARA algorithm performs well, significantly better than BTLBO and MI-LSFS. This indicates that OBL + ARA has extremely high robustness and performance stability in different tasks, demonstrating superior advantages in both specific and widely applied task categories.

**Table 2:** Comparison of different methods.

Method		Mean value	Variance	BTLBO	MI-LSFS	OBL + ARA
Wrestling class	top@3	0.33	0.33	0.1	0.2	0.66
	top@5	0.4	0.2	0.2	0.4	0.66
	top@10	0.7	0.7	0.3	0.5	0.8
Racing class	top@3	0	0	0	0.1	0.7
	top@5	0.2	0	0.1	0.2	0.7
	top@10	0.4	0.4	0.2	0.4	0.8
Skills class	top@3	0.33	0	0.2	0.3	0.6
	top@5	0.4	0.2	0.3	0.4	0.7
	top@10	0.3	0.3	0.3	0.4	0.7
Modern school class	top@3	0.33	0.33	0.2	0.3	0.66
	top@5	0.4	0.6	0.4	0.4	0.7
	top@10	0.5	0.6	0.5	0.4	0.8

### Performance of the ARA optimised based on the reverse learning mechanism with the effect of motion evaluation

Due to the existence of flow field factors, the movement direction of water droplets in the flow field is related to the flow field factors, which have a great influence on the overall and local optimisation seeking ability of the flow field. Aiming at this problem, the eye, based on the existing research, realises the adaptive adjustment to the difference of raindrop potential energy by introducing the adaptive flow field coefficients, so as to achieve the effective detection of the raindrop field. A test function is randomly selected for 1000 iterations, and 100 of them are selected, and the simulation obtains the variation graph of$\tau$

**Fig. 9:**
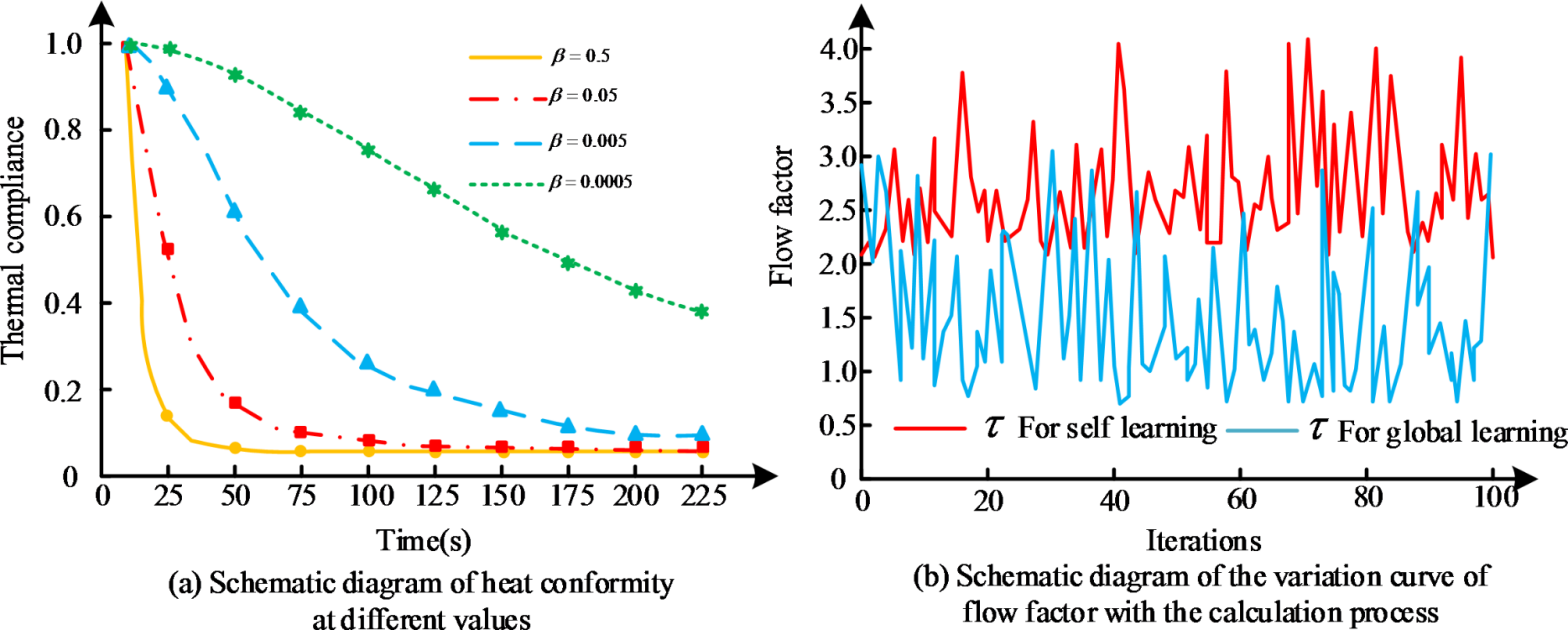
Schematic diagram of thermal consistency for different values and flow coefficient variation curve with calculation process.

**Fig. 10:**
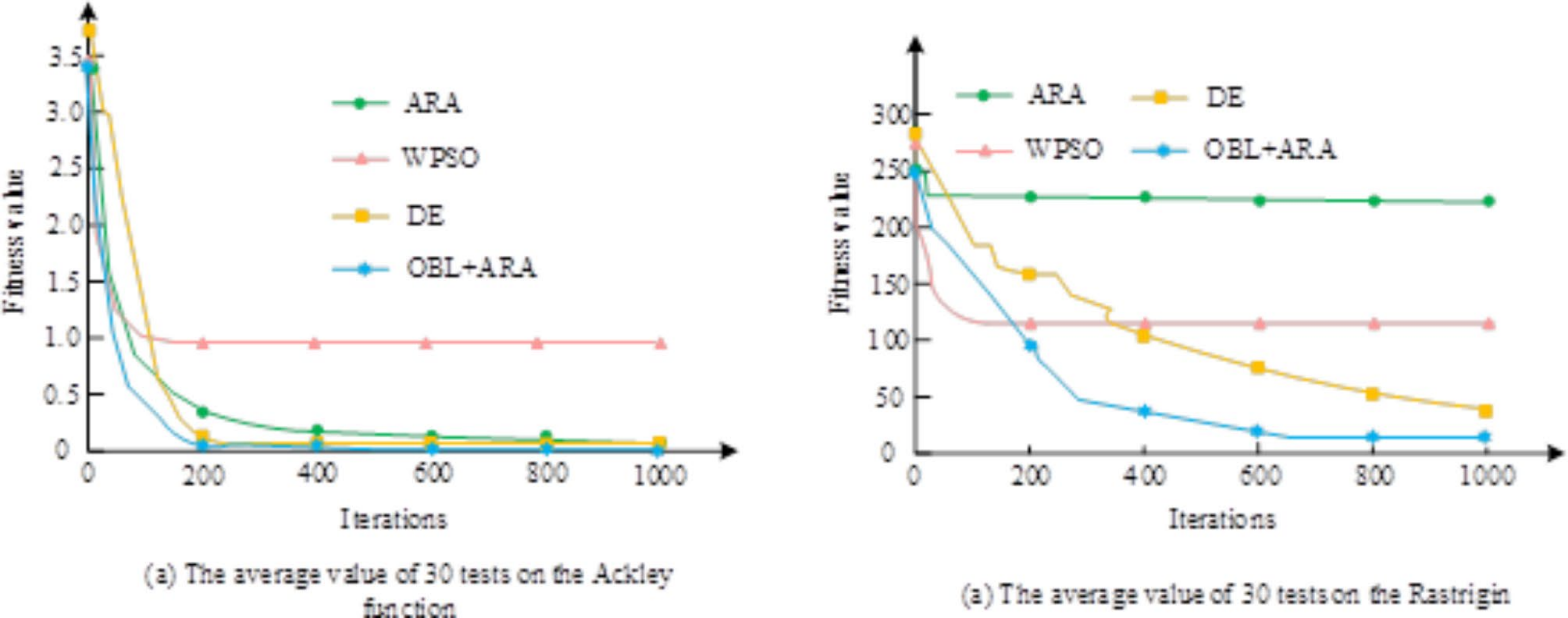
Convergence curves of four algorithms on test functions.

In Fig. 9(a), which represents a schematic diagram of the thermal conformance and a schematic curve of the variation of the flow coefficients with the computational process for different values, the larger$\beta$ is, the more sensitive it is to the thermal conformance, and the more accuracy the parameter needs to achieve. Where the optimum is reached when$\beta =0.005$. In Fig. 9(b), the two types of flow factors are in a complementary state, which is in line with the expectations of the study for them. In the simulation tests, ARA, dynamically weighted PSO, and differential evolutionary algorithm are used to achieve the optimal operation under the same conditions and compared with the three algorithms mentioned above and their performance is examined. The convergence curves of the average fitness values obtained by subjecting the four algorithms to 30 experiments on different test functions Ackley and Rastrigin test functions are shown in Fig. 10.

Figure 10 (a) and 10 (b) show the performance of four algorithms on two different function optimization problems, Ackley function and Rastrigin function, respectively. In Fig. 10 (a), the OBL + ARA algorithm exhibits the fastest convergence speed and the lowest fitness value, and is the best performing among the four algorithms. The ARA algorithm performs well in the early stages of iteration, but its final convergence value is not as good as OBL + ARA. The DE algorithm decreases rapidly in the initial stage, but the final result is slightly inferior to OBL + ARA. However, the WPSO algorithm showed almost no optimization effect throughout the entire iteration process. In Fig. 10 (b), the OBL + ARA algorithm also demonstrated the best optimization effect and convergence speed, while the ARA algorithm, although rapidly declining in the initial stage, still had a high fitness value in the end. The DE algorithm also showed a rapid downward trend in the initial stage, but the final effect is still not as good as OBL + ARA. The performance of WPSO algorithm on Rastrigin function is still poor, with no significant optimization effect. Overall, the OBL + ARA algorithm has demonstrated excellent robustness and adaptability in both test functions, especially when dealing with complex multimodal functions, where its advantages are more pronounced.

**Fig. 11:**
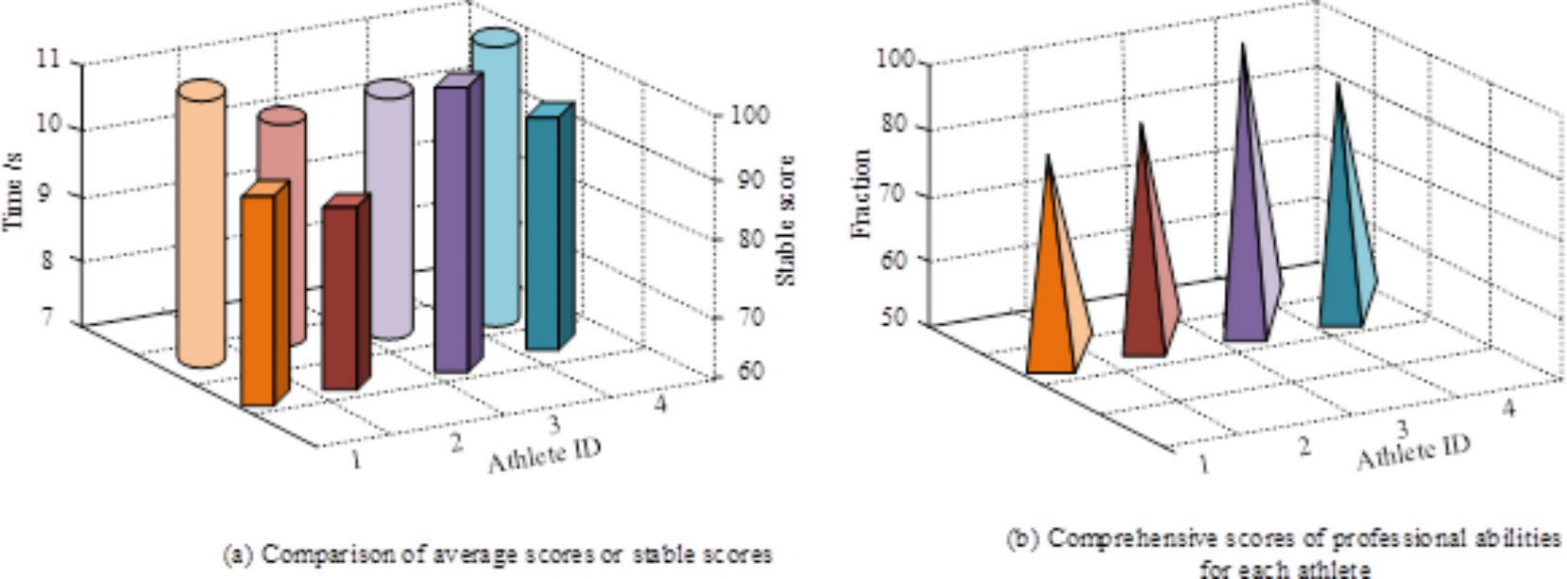
Analysis of the actual application effect of the proposed model.

Figure 11 shows the analysis results of the proposed model on the daily training data of a certain university athletics team. Figure 11 (a) shows the average score and stability score of the results of the four athletes in the 100 m sprint analyzed by the proposed model. The cylinder represents the average performance of different athletes, while the rectangle represents the stability of performance for different athletes. From the graph, it can be seen that athlete 2 has the best average sprint performance, but his performance stability is poor, and he is prone to poor form leading to low scores during the competition. And athlete 3’s average sprint score is only second to athlete 2, but the athlete’s stability score is relatively high. Therefore, overall, athlete 3’s professional level is higher than athlete 2. Figure 11 (b) shows the overall scores of four athletes analyzed by the proposed model. From the results in Fig. 11 (b), it can be seen that athlete 3 has the highest overall score, thus verifying the output of Fig. 11 (a).

## Reach a verdict

Currently, most SDM techniques work on efficient feature extraction and establishment of primary sports data, rather than simply relying on data statistics. The research organically integrated ML and DM, and explored RF-FSA methods for SDM analysis to achieve effective mining and analysis. A RF evaluation method for studying motion effects, using feature extraction algorithms to study the impact of motion effects, using IG indices to rank the importance of features, and achieving accurate evaluation of motion effects. The experimental results show that OBL + ARA performs well on various sports datasets, and this algorithm has great potential in practical applications such as sports performance evaluation and training optimization. In addition, the OBL + ARA algorithm can maintain high performance when processing different types of motion data. The OBL + ARA algorithm performs well in various indicators. On the training set, its accuracy and F1 score are significantly higher than the other two algorithms, reaching 0.833 ± 0.019 and 0.837 ± 0.020, respectively. In the same test set, the accuracy and F1 score of OBL + ARA remained the highest, at 0.854 ± 0.020 and 0.864 ± 0.021, respectively, indicating that the algorithm has strong generalization ability. Whether it is competition, skill, or physical training data, the OBL + ARA algorithm is effective in all aspects top@N The performance is excellent in both wrestling and modern school tasks, with a maximum of 0.8, which is significantly better than the other two compared algorithms. The OBL + ARA algorithm performs well and is suitable for various sports scenarios. Although the OBL + ARA algorithm has shown outstanding performance in research, there are still some limitations. Firstly, the complexity of the algorithm is high and the computational cost is relatively high, which may limit its application in resource constrained environments. Future research can attempt to improve the computational efficiency of algorithms through technologies such as parallel computing or distributed processing.

## Data Availability

The datasets used and/or analysed during the current study available from the corresponding author on reasonable request.
